# Thickness of the abdominal wall and pelvic floor dysfunctions in men who practice crossfit vs no crossfit: An observational study

**DOI:** 10.1371/journal.pone.0296595

**Published:** 2024-07-29

**Authors:** Carla Isabel Courtaut García, María Mateos Noblejas, Carlos Romero Morales, Beatriz Martínez Pascual

**Affiliations:** 1 Universidad Europea de Madrid, Villaviciosa de Odón, Madrid, Spain; 2 Faculty of Sport Sciences, Universidad Europea de Madrid, Villaviciosa de Odón, Madrid, Spain; 3 Faculty of Physical Activity and Sports Sciences, Department of Physiotherapy, Podiatry and Dance, Universidad Europea de Madrid, Villaviciosa de Odón, Madrid, Spain; Iran University of Medical Sciences, ISLAMIC REPUBLIC OF IRAN

## Abstract

**Introduction:**

The objective of this study is to compare the thickness of the transverse abdominis, internal oblique, external oblique, rectus abdominis, and rectus abdominis distance, the quality of life (SF-36), the presence of chronic pelvic pain (CPPQ-Mohedo), and sexual dysfunction (IIEF) in men who practice CrossFit^®^ versus men who do not.

**Design, setting, participants, and main outcome measures:**

Sixty-four healthy men with an average age of 37.19 were recruited at a private sports club and divided into two groups for this cross-sectional observational study. Additionally, participants completed the CPPQ-M, IIEF, and SF-36 questionnaires.

**Results:**

Significant differences were found in the thickness of the internal oblique at rest (p = 0.018, d = 0.61), which was greater in the CrossFit^®^ group. In the SF-36 quality of life questionnaire (p = 0.05, d = 0.50), the CrossFit^®^ group also obtained a higher score.

**Conclusion:**

CrossFit^®^ improves the quality of life and self-esteem of the participants, in addition to increasing the thickness of the internal oblique. Neither more chronic pelvic pain nor more erectile dysfunction was observed in the CrossFit^®^ group.

## Introduction

CrossFit^®^ is a high-intensity functional training modality, also known as HIFT [1]. This discipline was conceived as a combination of constantly varied functional exercises with a competitive component, integrating disciplines such as weightlifting, athletics, or gymnastics [2].

This training method has demonstrated multiple benefits, including improvements in cardiovascular and respiratory capacity, muscular endurance, power, and agility [3, 4]. However, it is also associated with the potential risk of musculoskeletal injuries. The injury rate is relatively low compared to other disciplines, with 3 ± 5 injuries per 1000 hours of training, showing an incidence similar to that of rugby or weightlifting [5, 6].

The etiology of CrossFit^®^ injuries may stem from advanced technical level requirements. Depending on the proposed work scheme, Incomplete rests are commonly employed in tandem with the sequential arrangement of circuit-type exercises and multi-joint components, prioritizing high intensity. These components can lead to early fatigue, oxidative stress, a greater perception of effort, and the execution of unsafe movements [7].

### Pelvic floor

The abdominal-pelvic region should be understood as a functional unit, normal tone in the transversus abdominis (TrA), pelvic floor and diaphragm muscles and physiological curvatures of the spine [8].

One of the functions of the TrA is to maintain the statics of the lumbo-pelvic region and the erector spinae musculature. Another function of this muscle is to perform co-contraction with the internal oblique (IO) and the pelvic floor musculature [9–11] mainly in load transfer movements and when intra-abdominal pressure (IAP) increases [4] such as in running and jumping [12]. All of these exercises are involved in the dynamics of CrossFit^®^. Unfortunately, an effective co-contraction, pre-contraction, or simultaneous contraction of the pelvic floor is not always observed during the increase of IAP [13].

During strains such as those described, the pelvic viscera moves backward and downward as a whole. This mobility is cushioned by the tone of the pelvic floor muscles, the sacral and coccyx joints, and the fasciae. If this displacement is too great, the ligaments and fasciae become excessively tense, causing an overload of the pelvic floor muscles, favoring their hypotonia and diminishing their support function [8, 14–16].

On the other hand, it is worth noting the importance of IAP in heavy-load exercises. The coordinated activation of the abdominal-lumbopelvic musculature ensures spinal stability and regulates IAP [17].

An IAP that increases during weight-bearing exertion will exert an upward force on the diaphragm and a downward force on the pelvic floor musculature. Although the literature is sparse, if the musculature is not able to withstand such increased pressure, it could be a factor in causing pelvic floor disturbances [18].

### Dysfunctions

Chronic pelvic pain is a musculoskeletal disorder that occurs in the lower abdomen, pelvis, or pelvic structures, with a duration of at least 3–6 months and occurs continuously or intermittently [13, 19]. It is associated with impaired quality of life, and although its prevalence is higher in women, it is also notable in men (15.6%) [20, 21]. It should be noted that it is also observed in athletes.

On the other hand, sexual dysfunction also predominates as pelvic floor dysfunction in both sexes [22]. According to the ICS (International Continence Society), sexual dysfunction constitutes the abnormal sensation and/or function experienced during sexual activity [23]. Associated with high volumes of physical activity, hypogonadism can occur, in which the basal testosterone level decreases and can be associated with a decrease in sexual activity and capacity [22].

Women who engage in high-intensity exercise are at greater risk of pelvic floor dysfunction than those who do not [13, 16]. In CrossFit^®^ in particular, there are studies focusing on female urinary incontinence [24–27]. However, few studies sample men who engage in high-intensity sports associated with dysfunction of the abdominopelvic region [28].

To date, there is a lack of evidence that assesses abdominal musculature and pelvic floor dysfunctions in men who practice CrossFit^®^, while there are studies evaluating pelvic floor dysfunctions in women doing CrossFit^®^ compared to sedentary women [26].

The primary objective of this study is to compare the thickness of the abdominal wall musculature in men who engage in CrossFit^®^ with men who do not participate in CrossFit^®^ but perform strength exercises without the high intensity component in the gymnasium.

Secondary objectives include evaluating the presence of pelvic pain, potential sexual dysfunction, and quality of life among these subjects.

## Methodology

### Study design

This cross-sectional observational study was conducted adhering to the STROBE (Strengthening the Reporting of Observational Studies in Epidemiology Statement) guidelines [29].

### Participants

A sample of 64 subjects was recruited from a private sports club (Republic Space) in Madrid and divided into two groups: men practicing CrossFit^®^ and men that perform strength exercises without the high intensity component.

### Inclusion criteria

Participants included men who had been actively involved in the specified physical activity (CrossFit^®^ or strength exercises) for at least six months and attended sessions at least three times a week [30].

### Exclusion criteria

Exclusion criteria encompassed individuals with a history of pelvic floor training in the previous year [31], prior pelvic floor surgery, trauma to the pelvic region, or inflammatory bowel disease [32].

### Sample size calculation

The G Power program (3.1) was utilized to calculate the sample size. A required sample of 64 subjects was determined to compare means between two independent groups, using t-tests, with a power of 0.88, an effect size of 0.8, and an α error of 0.05.

### Variables

The study measured several dependent variables using ultrasound:

Thickness of the TrA, IO, EO, and RA in centimeters, both during contraction and at rest and DIR in centimeters, both at rest and during contraction.

Additionally, subjective assessments were conducted using validated questionnaires for:

Pelvic pain measurement, erectile function assessment and evaluation of quality of life.

The independent variable in this study was the type of physical activity. It was treated as an inter-participant factor with two levels: CrossFit^®^ and strength exercises.

These classifications allowed for the comparison of the impact of different types of physical activities on the measured dependent variables.

### Procedure

#### Participant recruitment and informed consent

This study was approved by the research ethics committee of the University Europea of Madrid. (CIPI/22.018). We followed and respected the ethical standards of the Declaration of Helsinki [33].

The sports center staff provided a list of volunteers for the study. The recruitment period for this study was from the 28^th^ of March of 2022 to the 30^th^ of June of 2022. All participants were thoroughly informed about the study’s objectives and procedures. Prior to participation, each individual signed an informed consent form in paper. A paper survey, encompassing anthropometric data, sports activity duration, and information on previous pathologies related to inclusion and exclusion criteria, was then completed by each participant.

#### Questionnaire distribution

Subsequently, participants scheduled a date at the gym facilities. Upon arrival, one of the researchers anonymously handed them three paper questionnaires. Participants filled out these questionnaires, assessing pelvic pain, erectile dysfunction, and quality of life, using a pen before undergoing ultrasound measurements.

#### Questionnaires used

1. To assess pelvic pain, the CPPQ-Mohedo Spanish version from the University of Málaga questionnaire was employed (sensitivity and specificity: 0.968; reliability: Cronbach’s alpha, 0.75) [34].
2. For the evaluation of possible erectile dysfunction, the International Index of Erectile Function (IIEF-5) Spanish version was utilized [35] (reliability ICC 0.924. Convergent validity was excellent for the IIEF-5 with a correlation of r = 0.923) [36].
3. Finally, participants completed the SF-36v2^®^ Health Survey Spanish version quality of life questionnaire [37], with substantial evidence for reliability (Cronbach´s alpha greater than 0.85, reliability coefficient greater than 0.75 for all dimensions except for social functioning) and for construct validity in terms of distinguishing between groups with expected health differences [38].

#### Ultrasound measurements

Another researcher conducted ultrasound measurements of the abdominal region using the CHISON ultrasound scanner model SonoBook 9 with the following specifications: frequency of 10.2 MHz, IPS 53, D/P 110/0, GN 125, AP 100%, D 3.94 cm, and normal X-con.

#### Measurement protocol

Ultrasound measurements were conducted following Whittaker’s protocol [39, 40]. Participants were positioned supine with flexed hips and knees, maintaining relaxed legs. Muscle thickness was obtained by measuring the perpendicular thickness between the superficial and deep layers of fascia. Two images were collected at the end of the expiratory time during relaxed breathing after three consecutive breaths. The average of both measurements was recorded, with the researcher consistently positioned on the same side of the stretcher.

#### Measurement of resting thickness

**Fig 1:**
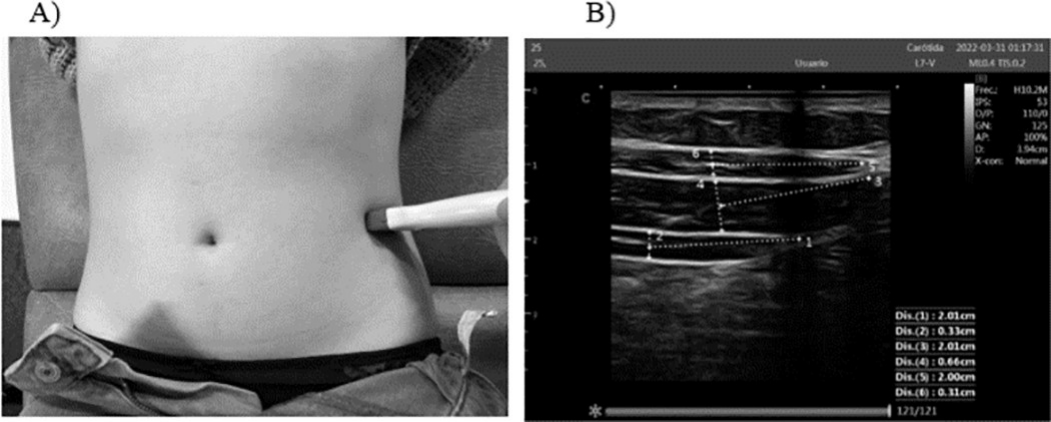
Thickness of the TrAb, IO, and EO. A) Probe placement. B) Measurements of TrA, IO and EO.

To measure the resting thickness of the TrAb, IO, and EO, the transducer was placed transversely at the midpoint between the iliac crest and the lower edge of the ribs on the anterolateral side of the abdomen. The thickness of TrAb and IO was measured along a reference line located 2 cm from the most medial point of TrAb [41] (Fig 1). Using the same transducer placement, a TrAb contraction was induced by performing the draw-in maneuver, instructing participants to draw the infraumbilical area inward and upward. Additionally, active elevation of the straight leg by 20 cm was requested to evaluate IO and EO.

#### Measurement for RA thickness

**Fig 2:**
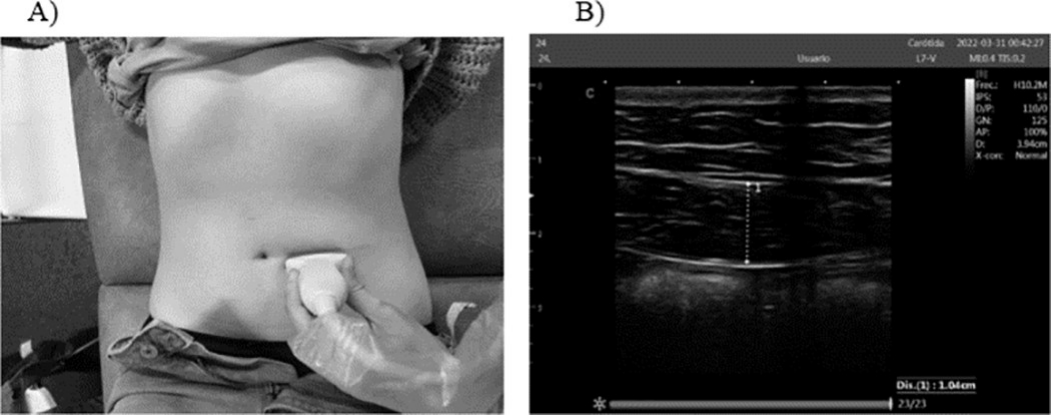
Measurement for RA thickness. A) Probe placement. B) Measurements of the RA.

The image was generated with the transducer positioned at the level of the umbilicus and laterally displaced from the midline until the cross-section of the muscle was centered (Fig 2).

This standardized approach ensured accurate and consistent measurements of RA thickness during the ultrasound assessment.

**Fig 3:**
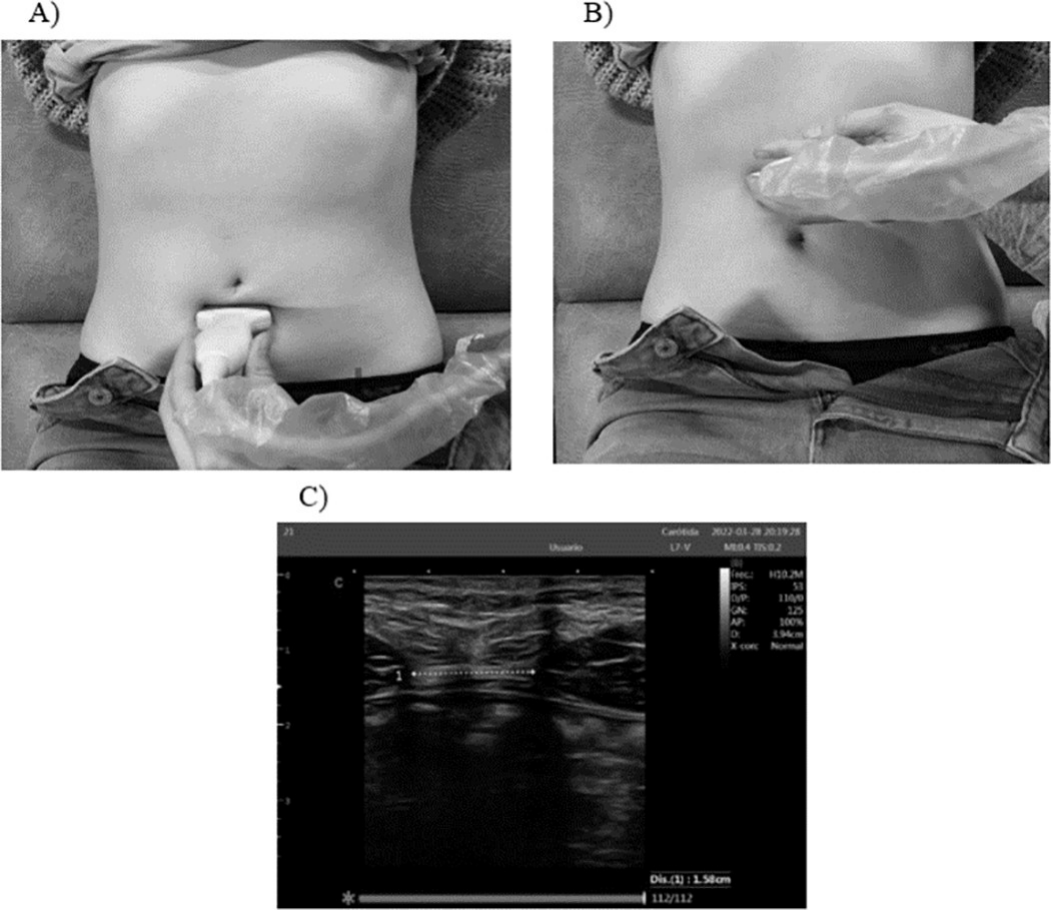
Measurement for DIR. A) Placement of the DIR probe 2cm below the umbilicus. B) Placement of the probe 3cm above the umbilicus. C) Measurements of the DIR.

Measurement for DIR: It was measured by placing the transducer transversely on the midline of the umbilicus, 3 cm above the umbilicus, and 2 cm below the umbilicus [42, 43] (Fig 3).

This specific positioning allowed for accurate assessment of the distance between different points of the rectus abdominis.

#### Assessment of contraction

To evaluate the contraction of both RA and DIR, participants were asked to elevate their upper body by 45° with respect to the stretcher. This standardized position ensured consistency in assessing the contraction of the specified abdominal muscles and distance measurements. consistency in assessing the contraction of the specified abdominal muscles and distance measurements.

#### Questionnaire evaluation

The questionnaires were individually evaluated using the official scoring criteria for each.

For the SF-36v2 questionnaire, eight health domains were assessed, including physical functioning, role-physical, general pain, bodily pain, vitality, social functioning, role-emotional, and mental health. The official scoring data sheet from the QualityMetric PRO CoRE Smart Measurement System version 2.1.1.18741 was employed for this evaluation.

The CPPQ-Mohedo questionnaire is divided into two dimensions (pain and quality of life) with a maximum score of 27. A score ≥ 6 on the questionnaire was considered indicative of chronic pelvic pain [21].

For the IIEF-5 questionnaire, five questions related to erectile function were posed. The score was calculated by summing the ordinal responses to the five items. The scoring categories were as follows:

- 22–25: No erectile dysfunction.
- 17–21: Mild erectile dysfunction.
- 12–16: Mild to moderate erectile dysfunction.
- 8–11: Moderate erectile dysfunction.
- 5–7: Severe erectile dysfunction [44].

### Statistical analysis

The thickness of the TrA, IO, EO, RA and the DIR in centimeters, both at contraction and at rest were measured as dependent variables. The quality of life, pelvic pain and erectile function, were also quantified.

The independent variable was the physical activity performed, this inter-participant factor had two levels: CrossFit^®^ and strength exercises. In this way, a contrast of means between the two groups was performed, testing the basic assumptions of normality using the Shapiro Wilk test and homogeneity of variances using the Levene test. However, since there were more than 30 participants in each of the groups, the central limit theorem gave us robustness to the violation of the normality assumption. In case of compliance with the assumptions, a t-test for independent samples was performed. In variables where the basic assumption of homogeneity (Levene’s) was not met, statistical analysis was performed as nonparametric data with the Welch’s test. The alpha level was 0.05 and the beta level was 0.12. The effect size was expressed by Cohen’s d, with values of 0.2, 0.5 and 0.8 for small, medium and large sizes, respectively. The R-based program Jamovi V.2.5 (www.jamovi.org) was used for statistical analysis and figure production.

## Results

Table 1 presents the mean and standard deviation of the anthropometric data for the entire sample. No statistically significant differences were observed in age, weight, height, and BMI (Body Mass Index) between the two groups. Likewise, there were no significant differences in the frequency of physical exercise, measured by the number of days per week participants engaged in either CrossFit^®^ or strength exercise. It is noteworthy that all 64 volunteers participated in the measurements, and each of them met the specified inclusion and exclusion criteria for the study. This uniformity in demographic and exercise-related factors enhances the reliability and comparability of the study results.

**Table 1:** Anthropometric data of the sample. Table prepared by the authors.

	CrossFit^®^ (n = 32)	Strength (n = 32)	p
Age	36.78 (±9.63)	37.59 (10.62)	0.843
Weight	80.07(10.36)	81.49 (11.01)	0.597
Height	1.78 (0.07)	1.80 (0.05)	0.098
BMI	25.33 (2.47)	25.06 (2.92)	0.689
Weekdays	4.53 (1.05)	4.56 (1.41)	0.920

Shown: Mean (±standard deviation), p-value.

BMI = body mass index

Table 2 shows the descriptive statistics of all the variables measured.

**Table 2:** Descriptive statistics, p value and effect size of transverse abdominis, internal oblique, external oblique, rectus abdominis muscle thickness, rectus distance, pelvic pain, erectile function and quality of life by group. Table prepared by the authors.

	Crossfit^®^(n = 32)	Strength (n = 32)	p	Effect size
TrAb R	0.45(±0.14) IC 95%: 0.40–0.50	0.40(±0.11) IC 95%: 0.36–0.44	0.134	0.38
TrAb C	0.65(±0.18) IC 95%: 0.59–0.71	0.61(±0.18) IC 95%: 0.55–0.67	0.387	0.22
IO R	0.96(±0.22) IC 95%: 0.89–1.04	0.85(±0.16) IC 95%: 0.79–0.90	0.018	0.61
IO C	1.13(±0.27) IC 95%: 1.04–1.22	1.03(±0.29) * IC 95%: 0.93–1.13	0.157	0.36
EO R	0.50(±0.13) IC 95%: 0.45–0.54	0.52(±0.13) IC 95%: 0.48–0.57	0.395	0.21
EO C	0.58(±0.14) IC 95%: 0.53–0.63	0.57(±0.17) IC 95%: 0.51–0.63	0.720	0.09
RA R	1.37(±0.28) IC 95%: 1.27–1.46	1.24(±0.30) IC 95%: 1.14–1.35	0.097	0.42
RA C	1.55(±0.36) IC 95%: 1.43–1.68	1.44(±0.34) IC 95%: 1.32–1.56	0.219	0.31
DIR SUPRA R	1.53(±0.88) IC 95%: 1.22–1.83	1.46(±0.84) IC 95%: 1.17–1.76	0.772	0.07
DIR SUPRA C	1.42(±0.90) IC 95%: 1.11–1.73	1.29(±0.79) IC 95%: 1.02–1.56	0.542	0.15
DIR INFRA R	0.40(±0.55) IC 95%: 0.21-0-59	0.26(±0.27) IC 95%: 0.16–0.35	0.209	0.32
DIR INFRA C	0.00(±0.38) Ɨ IC 95%: 0.11–0.50	0.06(±0.20) Ɨ IC 95%: 0.08–0.22	0.148 ƗƗ	0.37
SF-36	111.70(±6.96) Ɨ IC 95%: 108.11–112.55	110.06(±14.76) Ɨ IC 95%: 102.40–109.59	0.050 ƗƗ	0.50
CPPQ-M	0.97(±1.62) IC 95%: 0.41–1.53	1.69(±2.62) IC 95%: 0.78–2.59	0.191	0.33
IIEF	67.97 (±5.41) IC 95%: 66.06–69.87	65.84 (±11.38) IC 95%: 61.83–69.85	0.351	0.24

Mean (±standard deviation), Ɨ Median (±interquartile range), Student’s t test for independent samples, ƗƗƗ Welch’s t test. 95% CI = 95% confidence interval for the mean.

TrAb R: transverse abdominis at rest. TrAb C: transverse abdominis at contraction. IO R: internal oblique at rest. IO C: internal oblique at contraction. EO R: external oblique at rest. EO C: external oblique at contraction. RA R: rectus abdominis at rest. RA C: rectus abdominis at contraction. DIR SUPRA R: distance between the rectus abdominis above the umbilicus at rest. DIR SUPRA C: distance between the rectus abdominis above the umbilicus at contraction. DIR INFRA R: distance between the rectus abdominis below the umbilicus at rest. DIR INFRA C: distance between the rectus abdominis below the umbilicus at contraction. SF-36: quality of life questionnaire. CPPQ-M: pelvic pain questionnaire. IIEF: International Index of Erectile Function

**Fig 4:**
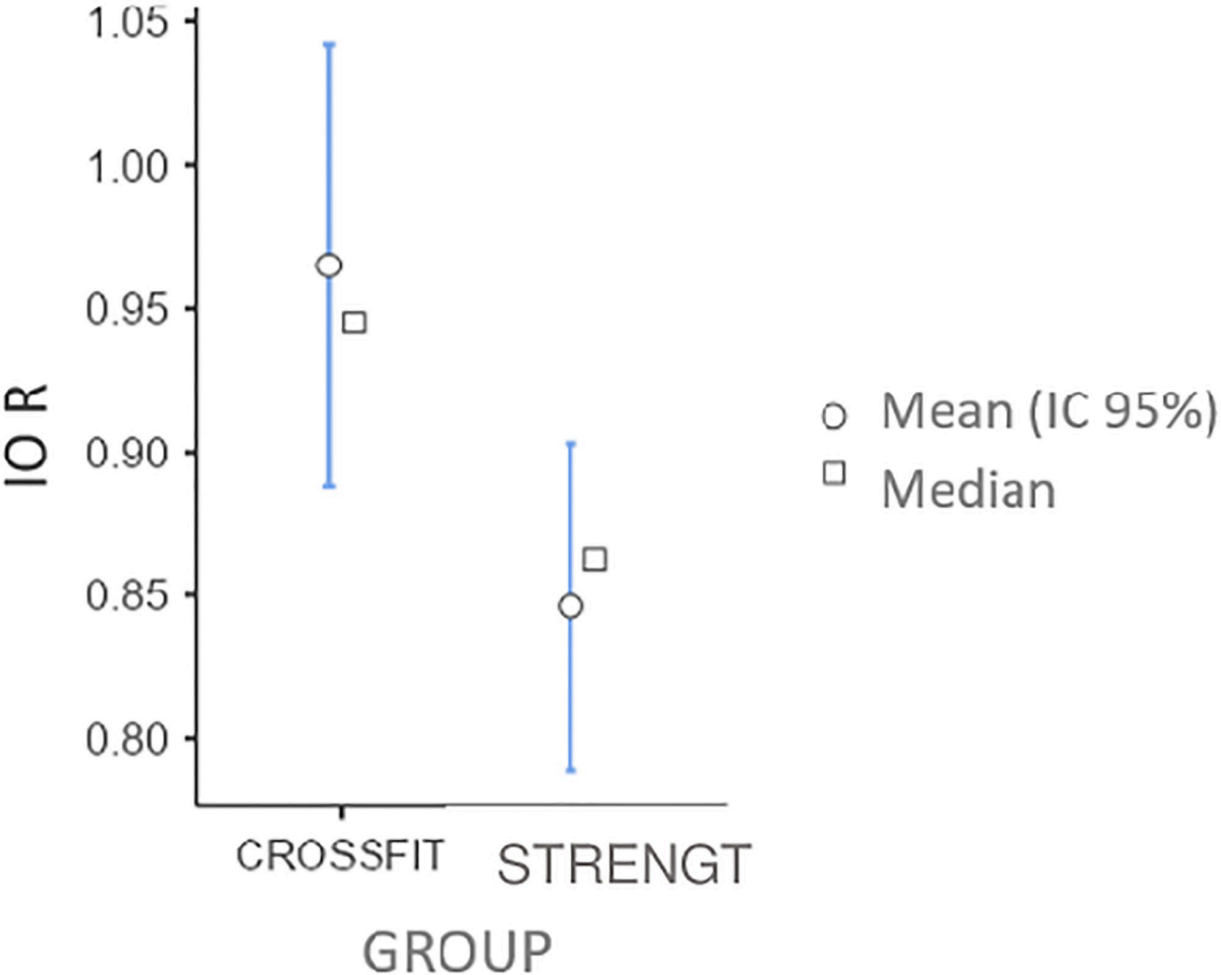
Differences between IO groups at rest. Significant differences in the CrossFit^®^ group.

The T-test for independent samples revealed that there were significant differences only in the resting IO measurement (Fig 4) t(62) = 2.43, p = 0.018, d = 0.61. The CrossFit^®^ group had a higher score.

In the rest of the dependent variables, no significant differences were observed between the two groups (p> 0.05).

## Discussion

The absence of previous studies measuring the thickness of the abdominal wall musculature or assessing pelvic floor dysfunctions in healthy men who practice CrossFit^®^ highlights a significant gap in the existing literature. In contrast, there have been several studies examining pelvic floor function in women engaged in CrossFit^®^ or other high-intensity sports.

This study contributes valuable insights by exploring a novel area of research, providing a foundation for understanding the impact of CrossFit^®^ on abdominal musculature and pelvic floor health in men. The lack of comparable studies in this specific population underscores the uniqueness and importance of the current investigation in addressing this research gap. The findings from this study could have implications for both the field of sports medicine and the broader understanding of the effects of high-intensity training on male pelvic health [24–26, 32, 45–47].

The recognition of physical activity as a potentially modifiable risk factor underscores the importance of understanding its relationship to pelvic floor dysfunctions, particularly given the significant burden experienced by women affected by these issues. Acknowledging physical activity’s role in pelvic health becomes crucial, as it opens avenues for preventive strategies.

As highlighted by DeLancey [48], prevention is a key aspect in mitigating the impact of pelvic floor dysfunctions. The notion that achieving a 25% prevention goal could spare more than 90,000 women per year from dysfunction emphasizes the potential public health impact of targeted preventive measures. This insight reinforces the need for comprehensive research, education, and interventions to address and reduce the prevalence of pelvic floor dysfunctions, emphasizing the importance of a proactive and preventative approach in women’s health.

The study conducted by Pisani et al. [32] focused on analyzing various pelvic floor dysfunctions in women engaged in CrossFit^®^. The findings indicated that anal incontinence, particularly gas incontinence, was the most frequent dysfunction (52.7%), followed by dyspareunia (48.7%), urinary incontinence (36%), vaginismus (2.5%), and prolapse (1.4%). This study contributes valuable information to the understanding of how specific dysfunctions manifest in the context of high-intensity physical exercise.

There is a body of evidence suggesting a potential link between high-impact physical exercise and the development of urinary incontinence symptoms, as observed in studies by Machado et al, Forner et al [26, 45] and Carvalhais, Natal Jorge, & Bø [49]. In contrast, evidence supports the idea that engaging in moderate physical activity may decrease the risk of developing urinary incontinence, as demonstrated by findings in the study by Townsend et al. [50].

These contrasting results underscore the importance of considering the intensity and type of physical activity in relation to pelvic floor health, emphasizing the need for nuanced approaches in understanding the impact of exercise on pelvic floor dysfunctions in women.

The existing evidence on pelvic floor dysfunctions in male athletes is notably limited, with current research primarily focusing on the effects of exercise following a radical prostatectomy, as indicated by studies such as Zachovajevienė, Šiupšinskas, Zachovajevas, & Milonas [51] & Milios, Ackland, & Green [52]. Despite this scarcity, there is acknowledgment that male athletes, particularly Olympic weightlifters, also experience pelvic floor disorders.

For instance, among Olympic weightlifters, urinary and anal incontinence have been reported with prevalence rates of 9.3% and 61.8%, respectively [53]. Alarmingly, more than 70% of these men did not comprehend the reasons behind their incontinence, and over half lacked knowledge on how to train the pelvic floor effectively. This emphasizes the need for increased awareness and education on pelvic floor health in male athletes.

The impact of pelvic floor dysfunctions in men who engage in sports extends beyond the physical realm, affecting them socially, psychologically, and economically, as highlighted by Dornan [54]. Despite the evident consequences, visibility and research attention on this subject remain limited, underscoring the importance of expanding research efforts in this area to better understand and address male athletes’ pelvic floor health.

The study acknowledges that sexual dysfunction may be associated with high volumes of physical activity, although there is no previous evidence on this dysfunction specifically in CrossFit^®^ athletes. Interestingly, the current study did not find significant differences in erectile dysfunction between the two groups, indicating a comparable prevalence of this issue among men practicing CrossFit^®^ and those engaging in strength exercises.

Comparative findings from other sports disciplines have revealed higher rates of erectile dysfunction in men who practice cycling [55]. Additionally, studies have highlighted low testosterone levels and indicators of erectile dysfunction associated with concussions suffered during sports practice, specifically in former American soccer players [56]. There have also been reports of transient hypogonadism in a male kickboxer [57].

These comparisons underscore the complexity of the relationship between physical activity, testosterone levels, and erectile function, indicating that the impact may vary across different sports disciplines. The absence of significant differences in erectile dysfunction in CrossFit^®^ athletes in the current study contributes to the understanding of sexual health in this specific athletic context.

In terms of chronic pelvic pain, the present study did not find significant differences between men who practiced CrossFit^®^ and those who did not. This aligns with the findings of Mohedo et al. [21], who reported a prevalence of pelvic pain in men at 15.6%. Moreover, our results are consistent with the observations of Zhang et al. [58] and Franco et al. [59], suggesting that regular exercise can reduce the risk of chronic pelvic pain, irrespective of exercise intensity.

Regarding abdominal muscles, the study revealed a significant difference in the thickness of the IO muscle at rest, with greater thickness observed in the group of men who performed CrossFit^®^. This finding is reminiscent of Sitilertpisan et al. [60], who found differences in the thickness of the TrA and IO among women practicing weightlifting, with the IO showing more pronounced differences. This could be attributed to the role of the IO in transferring loads between the pelvis and thorax, particularly when balancing external loads during weightlifting.

The hypertrophy of the IO in CrossFit^®^ practitioners may result from altered patterns in fast, repetitive movements, characteristic of this training modality. The literature generally supports the notion that the IO is more active than the RA in lifting exercises, with some variation in activation patterns among abdominal muscles, potentially influenced by different measurement techniques [61].

The existing evidence in this field, although limited, includes studies such as the one conducted by Gephart et al. [4], which analyzed IAP in women performing CrossFit^®^ in 2018. The study observed significantly higher IAP during specific exercises, such as double jumps, front squats, push-ups, and balls to the wall exercises. Moreover, IAP increased after multiple repetitions of back squats. These findings highlight that fatigue or changes in breathing may contribute to an elevation in IAP, which can potentially impact the pelvic floor musculature. Poor management of pressures during exercise, along with a lack or excess of abdominal tone, has been associated with pelvic dysfunction [62, 63]. Therefore, it underscores the importance of incorporating pelvic floor training in the exercise regimen of athletes.

On a broader note, numerous studies have demonstrated the positive effects of physical exercise on both psychological and physical health status. Additionally, correlations have been established between physical activity and variables such as mental health, stress, mood, anxiety, depression, self-efficacy, self-concept, and overall quality of life [64–66]. These associations underline the multifaceted benefits of regular physical activity, contributing not only to physical well-being but also to mental and emotional health.

According to the results of our study, the CrossFit^®^ group exhibited a higher quality of life compared to the group engaged in strength activity. This finding aligns with existing knowledge that high-intensity workouts, such as those characteristic of CrossFit^®^, offer benefits in various health conditions. For instance, research has shown positive outcomes in testicular cancer patients, postnatal urinary incontinence, and diabetes mellitus, with improvements in metabolic and quality of life outcomes, including mental and physical health components as measured through the SF-36 quality of life questionnaire [67, 68].

Consistent with our results, Gonzalez-Vallejo et al. [69] investigated the psychological well-being and quality of life of adults engaging in CrossFit^®^ three days a week, observing a significant improvement in these variables compared to a control group. Similarly, Ribeiro Neto, Magalhães, Walsh, & Bertoncello [70] interviewed official trainers of the CrossFit^®^ discipline, noting that they reported a good quality of life. This improvement could be attributed not only to the specific benefits of CrossFit^®^ but also to the active lifestyle of these professionals, with their direct influence on health.

As a sport activity, CrossFit^®^ has been associated with enhancing concentration, motivation, and self-confidence among participants. Additionally, it has been shown to reduce stress and foster a sense of teamwork [71]. These psychological and social aspects contribute to the overall well-being and quality of life experienced by individuals engaged in CrossFit^®^.

## Conclusion

The current available evidence, though limited, suggests that CrossFit^®^ as a high-intensity activity has positive effects on participants’ quality of life and self-esteem. Additionally, it was observed to increase the thickness of the IO. Importantly, the study did not find an increase in chronic pelvic pain or erectile dysfunction in the CrossFit^®^ group, indicating that CrossFit^®^ does not appear to have either beneficial or detrimental effects on the abdominal-lumbosacral-pelvic complex in these specific aspects.

It’s worth noting that more research in this area could contribute to a better understanding of the impact of CrossFit^®^ on various health parameters and abdominal muscle dynamics.

## Supporting information

S1 Checklist Human participants research checklist. (DOCX)
